# Antifungal Properties of *Chenopodium ambrosioides* Essential Oil Against *Candida* Species

**DOI:** 10.3390/ph3092900

**Published:** 2010-09-01

**Authors:** Marie Stéphanie Goka Chekem, Paul Keilah Lunga, Jean De Dieu Tamokou, Jules Roger Kuiate, Pierre Tane, Gerard Vilarem, Muriel Cerny

**Affiliations:** 1 Laboratory of Microbiology and Antimicrobial Substances, Faculty of Science, University of Dschang, P.O. Box 67 Dschang, Cameroon; 2 ENSIACET, Laboratoire de Chimie Agro-Industrielle, 4, Allée Emile Monso, 31432 Toulouse Cedex 4, France; 3 Laboratory of Natural Products, Faculty of Science, University of Dschang, P.O. Box 67 Dschang, Cameroon

**Keywords:** *Chenopodium ambrosioides*, essential oil, antifungal, yeasts, fatty acids profile

## Abstract

The essential oil of the aerial part (leaves, flowers and stem) of *Chenopodium ambrosioides* was obtained by hydrodistillation and its chemical composition analyzed by GC and GC/MS, which permitted the identification of 14 components, representing 98.8% of the total oil. Major components were α-terpinene (51.3%), *p*-cymene (23.4%) and *p*-mentha-1,8-diène (15.3%). The antifungal properties of this essential oil were investigated *in vitro* by the well diffusion and broth microdilution methods. The *in vitro* antifungal activity was concentration dependent and minimum inhibitory concentration values varied from 0.25 to 2 mg/mL. The *in vivo* antifungal activity was evaluated on an induced vaginal candidiasis rat model. The *in vivo* activity of the oil on mice vaginal candidiasis was not dose-dependent. Indeed, all the three tested doses; 0.1%, 1% and 10% led to the recovery of mice from the induced infection after 12 days of treatment. The effect of the essential oil on *C. albicans* ATCC 1663 fatty acid profile was studied. This oil has a relatively important dose-dependent effect on the fatty acids profile.

## 1. Introduction

Candidiasis is an opportunistic infection caused by fungal species of the genus *Candida,* predominantly *Candida albicans. Candida* species are ubiquitous fungi that represent the most common fungal pathogens that affect humans. The growing problem of mucosal and systemic candidiasis reflects the enormous increase in the number of patients at risk and the increased opportunity that exists for *Candida* species to invade tissues normally resistant to invasion [1]. Several factors contribute to this situation, including immunodeficiency in HIV-infected persons that results in patients who are more susceptible to fungal infections. About 77% of immune-deficient patients’ deaths are caused by fungi, including *Candida* species [2,3]. About 75% of women must have had at least one episode of vulvo-vaginal candidiasis, which is considered the second most common form of vaginitis after bacterial vaginosis. during their lives [4]. It is also important to mention that drug-resistant *Candida* strains have been reported [5,6].

Commonly used drugs that eradicate fungal infections are the imidazoles and polyenes [7,8]. Polyene antifungal agents such as amphotericin B interact with membrane sterols resulting in production of an aqueous pore that leads to altered cellular permeability [9]. On the other hand, fatty acids are potent targets for some antifungal compounds since they form part of the major building blocks of fungal cells [9]. In addition to the fact that some of these drugs have relatively high toxic effects and are relatively expensive, their antifungal spectra are usually limited to a reduced number of fungal species [10]. 

In the recent years, there has been growing interest in antifungal essential oils from natural products, and interesting results have been obtained for azole-susceptible and -resistant *Candida* species with the essential oil of *Melaleuca alternifolia* (tea tree) [11]. The essential oil of *Chenopodium ambrosioides* is known to inhibit the growth of dermatophytes [12] and other filamentous fungi such as *Aspergillus*, *Fusarium* and *Colletotrichum* [13]. It equally possesses anti-aflatoxigenic, antimalarial and antioxidant properties [14] as well as antihelmintic and worm expelling activities [15]. To the best of our knowledge, the antifungal properties of this oil against yeast species are being reported here for the first time, with emphasis on the fatty acid profiles. The present work was thus designed to evaluate the *in vitro* and *in vivo* antifungal properties of *C. ambrosioides* essential oil on some human pathogenic yeast species and equally its effects on fatty acid profiles of *Candida albicans* in order to contribute to a possible standardization of this oil in the treatment of vaginal candidiasis.

## 2. Results and Discussion

### 2.1. Chemical composition of the essential oil

The hydrodistillation of the aerial part of *C. ambrosioides* gave a yellowish essential oil (0.12%). Fourteen components, all monoterpenes, were identified, with α- terpinene (51.3%), *p*-cymene (23.4%) and *p*-mentha-1,8-diene (15.3%) being the most abundant (Table 1).

**Table 1:** Chemical composition (%) of the essential oil of the aerial part of *C. ambrosioides.*

N°	Constituents	RI	%
1	α-Pinene	936	0.1
**2**	**α-Terpinene**	**1028**	**51,3**
**3**	***p*-Cymene**	**1035**	**23,4**
4	Limonene	1036	0,9
5	β-Phellandrene	1038	0,2
6	γ-Terpinene	1063	0,7
7	Dehydro-*p*-cymene	1096	0,1
8	L-Carvacrol	1128	0,1
**9**	***p*-Mentha-1,8-diene**	**1291**	**15,3**
10	Oxyde de piperitone	1302	0,4
11	Ascaridole	1305	0,7
12	Thymol	1332	0,2
13	Carvacrol	1340	0,3
14	Isoascaridole	1347	5,1

All constituents were identified by Gas Chromatography-Flame Ionization Detector and Gas Chromatography-Mass Spectrometry; RI: Retention Index.

These results were significantly different from those obtained by Tapondjou *et al.* [16] for an oil sample extracted from dry leaves of *C. ambrosioides* collected from the same geographical area. It is possible that during the drying process some of the chemical constituents underwent chemical transformations and disappeared. This may be the case with *p*-mentha-1,8-diene and some other constituents that were absent in the sample of Tapondjou *et al.* [16]. These differences can also be due to physiological variation, genetic factors and the evolution as well as the harvest time and period [17]. Also, from the results published so far, the concentration of the main constituents of *C. ambrosioides* oil varies considerably. The concentration of ascaridole, described as a quality indicator of this oil [15] was very low in Cameroonian, Nigerian [18] and Indian [19] samples. In contrast, ascaridoles appear to be the main constituents of the Brazilian [13], Togolese [20] and French commercial essential oil samples [21]. Although the chemical composition of the oil can be influenced by environmental factors, these disparities in results may also suggest the existence of chemotypes in *C. ambrosioides* plant species.

### 2.2. Antifungal properties

All the tested microorganisms were sensitive to the essential oil in the *in vitro* antifungal study and this activity was concentration-dependent (Table 2). MIC values varied from 0.25 to 2 mg/mL with *C. glabrata* and *C. guilliermondi* (MIC = 0.25 mg/mL) being the most sensitive while *C. albicans* ATCC 2091**(**MIC = 2 mg/mL) was the most resistant. *C. glabrata* presented the smallest MFC (0.25 mg/mL). However, this *in vitro* antifungal activity was lower than that of nystatin used as reference antifungal agent.

**Table 2:** Minimum Inhibitory Concentrations (MIC) and Minimum Fungicidal Concentrations (MFC) of essential oil of *C. ambrosioides* on *Candida* strains.

Microorganism	MIC		MFC	
	Essential oil (mg/mL)	Nystatin (µg/mL)	Essential oil (mg/mL)	Nystatin (µg/mL)
*C. albicans* ATCC 9002	1.00	1.00	2.00	2.00
*C. albicans* ATCC 2091	2.00	1.00	2.00	2.00
*C. albicans* ATCC 1663	1.00	1.00	2.00	4.00
*C. glabrata*	0.25	1.00	0.25	1.00
*C. guilliermondi*	0.25	1.00	0.25	2.00
*C. krusei*	1.00	1.00	2.00	4.00
*C. lusitaneae*	1.00	2.00	1.00	4.00
*C. parapsilosis*	0.50	0.50	1.00	1.00
*C. tropicalis*	1.00	0.50	1.00	1.00

Tabulated values are the means of three trials which did not show any variation.

Many other biological activities of the essential oil of *C. ambrosioides* have been reported. It possesses antifungal activities on dermatophytes [12] and other filamentous fungi such as *Aspergillus*, *Fusarium* and *Colletotrichum* [13]. This is however the first time the antifungal properties of this oil are being investigated *in vitro* and *in vivo* on *Candida* species with emphasis on its effect on fungal fatty acid profiles. This antifungal activity varied with the fungal species and for the same species with the strain. These variations may be due to genetic differences between the microorganisms.

**Figure 1:**
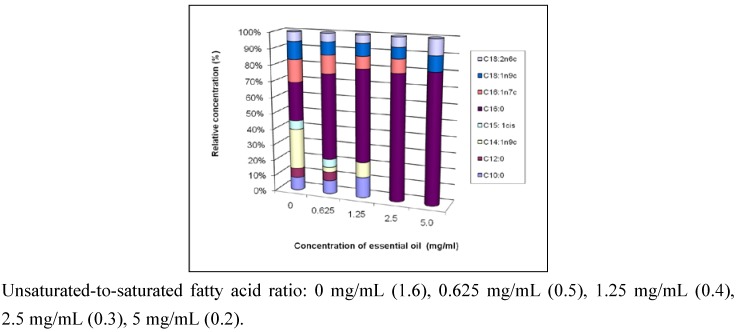
Effect of the essential oil of *C*. *ambrosioides* on the relative proportion of fatty acids in *C. albicans* fatty acid profiles.

The effect of the essential oil on fatty acid profile reveals that the global fatty acid quantity decreased with increasing oil concentration. The relative concentration of each fatty acid in the microorganisms was also dependent on the oil concentration used, with some fatty acids being more affected than others, while some even disappeared (Figure 1). Fungi subjected to a concentration of 0.0625 mg/mL synthesized pentadecanoate and pentadecenoate, but as the oil concentration increased these fatty acids were no longer present in the fungal extract. These results suggest that the microorganism fights against the oil by using these fatty acids to re-enforce the membrane impermeability, but the higher oil concentration seems to inhibit the enzymes involved in their synthesis. 

Fatty acids are major building blocks of cell membranes [22]. In this study, we found that the ratio of unsaturated-to-saturated fatty acids decreases from 1.6 to 0.2, associated with a shift from C_18_ (oleate and linoleate) to C_16_ (palmitate) (Figure 1). Similar results have been reported with naftifine and tolnaftate which are inhibitors of ergosterol synthesis [9]. Thus, it is probable that the essential oil of *C. ambrosioides* elicites a response against *Candida* by impairing ergosterol synthesis. This suggests that the oil may affect membrane fluidity and permeability and consequently protein function, increasing possibly the uptake of undesired substances by fungal cells. It is the case with chitosan on sensitive fungi for which more polyunsaturated fatty acids are produced compared to non-sensitive, suggesting that their permeabilization by chitosan may be dependent on membrane fluidity [11,22]. This perturbation of membrane integrity and architecture may distort membrane function [23,24] and may explain the antifungal activity of the essential oil. Membrane saturation may play an important role in the effectiveness of antifungals [25].

**Figure 2:**
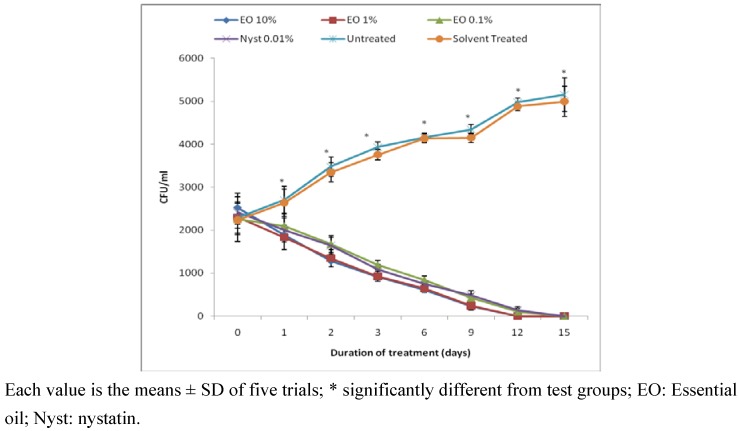
Evolution of healing effect of the essential oil of *C. ambrosioides* on vaginal candidiasis model in rat as a function of oil concentration.

The therapeutic effect of the oil on mice vaginal candidiasis model showed that the infection was established in all experimental animals two days after inoculation with *C. albicans*. The treatment of these animals with oil solutions at doses of 0.1%, 1% and 10% (weight/volume) induced an important time-decrease in the number of yeast colonies in vaginal fluid of infected animals (Figure 2). This antifungal effect was not dose-dependent *in vivo*. All the animals that received the three essential oil doses got healed within 12 days of treatment as those treated with nystatin (0.01%). The healing effect of *C. ambrosioides* oil was more rapid compared to that reported by Mondello *et al*. [11] for the essential oil of *Melaleuca alternifolia* which at a dose of 1% (weight/volume) healed mice infected with *C. albicans* after 23 days of treatment. Also, tepinen-4-ol, the main bioactive component of this oil showed good activity in controlling *C. albicans* vaginal candidiasis.

## 3. Experimental

### 3.1. Plant material, oil extraction and chemical composition

The aerial part (leaves, flowers and stem) of *C. ambrosioides* was collected on the Dschang University Campus in August 2008 and its taxonomic identification performed at the National Herbarium of Yaounde/Cameroon. A voucher specimen was deposited in the Herbarium of the Biochemistry Department of the University of Dschang. The essential oil was obtained by hydrodistillation for 5 h of fractions of fresh aerial plant material (3000 g) using a Clevenger-type apparatus. The oil obtained was dried over a column of anhydrous sodium sulphate and stored in dark bottles at +4 ºC until analysis.

The chemical composition of the essential oil of *C. ambrosioides* was determined by GC and GC/MS. The GC analysis was carried out using a Hewlett-Packard model 5890 apparatus fitted with a DB-5 fused silica column (30 m × 0.32 mm, 0.25 µm film thickness). The oven temperature was programmed from 50 to 200 ºC at 5 ºC/min. The injector and detector (FID) temperatures were fixed at 200 ºC and 250 ºC respectively.

The GC-MS analysis were carried out using an Agilent 6890N Network GC system/5975 Inert x L Mass selective Detector at 70 eV and 20 ºC. The GC column was a CP- Sil 8 CB LB, fused silica capillary column (0.25 mm × 30 m, film thickness 0.25 µm). Helium was used as carrier gas at a flow rate of 1.6 mL/min. The injector port was maintained at 250 ºC; the oven temperature was programmed at 5 ºC/min from 50 ºC. The split ratio was 1:100. One microliter of pure essential oil was injected into the GC and GC-MS apparatus. The retention indices were calculated relative to C8-C19 n-alkanes. The constituents were identified by comparison of their retention indices with those of the literature (Pherobase-Kovats Indices) and by matching the mass spectra data with those stored in NIST05 and Wiley237 database libraries.

### 3.2. Antifungal assay

#### 3.2.1. Fungi strains and culture media

The microorganisms used in this study were *C. albicans* ATCC 9002, *C. albicans* ATCC 2091, *C. albicans* ATCC 1663, *C. parapsilosis* ATCC 22019, *C*. *tropicalis* ATCC 750, C. *krusei* ATCC 6258, *C. parapsilosis* ATCC 22019 and *C. lusitaniae* ATCC 200950. The *C. albicans* ATCC 1663 strain was used for the *in vivo* studies and for the determination of the effect of the oil on fatty acids profile. *C. albicans* is the major *Candida* species causing oral and vaginal candidiasis and systemic infection which has frequently recurred in the immunocompromised patients [26]. 

Sabouraud Dextrose Agar (SDA) culture medium (Liofilchem Laboratories) was used for the determination of inhibition zone diameters and for sub-culturing during minimum fungicidal concentration determination. Sabouraud Dextrose Broth (Conda) was used for the determination of minimum inhibitory and fungicidal concentrations and for the study of the oil’s effect on fungal fatty acid profiles.

#### 3.2.2. Determination of minimum inhibitory concentrations and minimum fungicidal concentrations

The *in vitro* antifungal properties of *Chenopodium ambrosioides* essential oil were measured using the microdilution method [27]. The inoculum suspensions were prepared from 24 h colonies grown on an agar plate of SDA and inoculated into saline physiological solution to yield a 0.5 McFarland turbidity standard suspension corresponding to about 1.5 × 10^8^ CFU/mL. From these prepared suspensions, other dilutions with saline physiological solution were done to give a final concentration of 2 × 10^5^ CFU/mL. Stock solution (160 mg/mL) of *Chenopodium ambrosioides* essential oil dissolved in Tween 80 (5%) was two-fold serially diluted to give 80–0.625 mg/mL concentration range. 

Ninety six well sterile plates (IWAKI) were filled with Sabouraud Dextrose Broth (185 µL). Ten microliters of essential oil solutions were introduced in each well and 5 µL (2 × 10^5^ CFU/mL) of yeast suspension were added. A growth well (broth and inoculum) and a sterility control well (broth only) were included in each panel. The plates were then incubated at 35 ºC for 48 h on a rotating shaker (Titertek) at 300 rpm. Minimum inhibitory concentration (MIC) was determined as the smallest oil concentration at which no increase in visual turbidity was observed [27]. Minimum fungicidal concentration (MFC) was determined by plating 10 µL from each negative well and from the positive growth control on SDA. MFC was defined as the lowest concentration yielding negative subcultures or only one colony [28]. All the experiments were carried out in triplicate. Nystatin served as reference antibiotic.

#### 3.2.3. Effect of *C. ambrosioides* essential oil on *C. albicans* fatty acid profiles

##### 3.2.3.1. Fungal culture and fatty acid extraction

*C. albicans* ATCC 1663 strain was used in this study. Five sets of three 100 mL conical flasks each were prepared and 50 mL of Sabouraud dextrose broth medium and essential oils at final crather Tween 20 at 1% concentration. Into each conical flask was introduced a 2 × 10^5^ CFU/mL suspension of *C. albicans* (400 µL). These preparations were incubated under constant mechanical shaking (Titertek) at 35 ºC for 48 h. The biomass was harvested by centrifugation at 1,500 rpm for 15 min. The sludge (yeast cells) was washed with methanol and fungal fatty acids extracted as below. The method described by the protocols of the MIDI chromatographic system was used for fatty acids extraction [29]. The procedure began by a saponification step using NaOH 3.75 M-methanol 50% (1/1, v/v, 1 mL) and fungal biomass (1 mg) in test tubes. This mixture was vortexed for 5 to 10 seconds, and heated in a boiling water bath for 5 min. Tubes were vortexed again for 10 seconds and returned to the boiling water bath for 25 min. The tubes were then cooled to room temperature and the methylation process followed by the addition of a 6.0 M solution of HCl/methanol (13/11, v/v, 2 mL). The homogenized mixture was heated at 80 ºC for 10 min and rapidly cooled using tap water. The methylated fatty acids were then extracted by adding hexane/methyl *tert*-butyl ether (1/1, v/v, 1.25 mL) and gently mixed by continuous immersion for 10 min. The topmost phase containing the methylated fatty acids was collected and washed with 0.3 M NaOH (3 mL). Two-thirds (2/3) of the organic phase was transferred into chromatographic vial for GC analysis.

##### 3.2.3.2. Fatty acid profile analysis

Hexane was removed from the samples by evaporation and then pure hexane (50 µL) was added to each vial. The analysis was carried out in triplicate in a Varian 3900 Gas Chromatograph equipped with a CP–Select CB for FAME fused silica WCOT column, 50 m long, 0.25 mm interior diameter, 0.25 µm film thickness. The oven temperature was programmed at 185 ºC for 40 min and 15 ºC/min up to 250 ºC and at 250 ºC for 10 min. Injection split was 1:100 and the sample volume injected was 1 µL. Detector and FID temperature were fixed at 250 ºC. The identification of individual FAME was done on the basis of their retention time relative to a mixture of standards FAME: capric acid, lauric acid, myristic acid, myristoleate, pentadecanoate, pentadecenoate, palmitate, palmitoleate, stearate, oleate, linolenate, arachidonic acid.

#### 3.2.4. Evaluation of therapeutic effect of *C. ambrosioides* essential oil

Pathogen-free female *albino Swiss* mice (6 weeks old) were used for the candidiasis experiments. These animals were bred in the animal house of the Faculty of Science of the University of Dschang. Animal experiments were carried out according to the guidelines provided by the Ethic Committee of the University of Dschang.

Thirty five mice previously acclimatized for one week were distributed into seven groups of five animals each. Animals were injected subcutaneously with 0.1 mL of a solution of oestradiol (0.5mg/mL) [4]. Animals were infected intravaginally using 10 µL of *C. albicans* suspension (1.5 × 10^8^ CFU/mL) [25]. The different groups were treated as follows:

- Group 1 received nystatin at 0.01% (positive control),
- Group 2 was infected and not treated (negative control),
- Group 3 was infected and received 10 µL of Tween 80 solution 1%,
- Group 4 was not infected and did not receive any substance,
- Group 5, 6 and 7 were infected and treated with 0.1, 1 and 10% of essential oil solutions (test groups).

Administration of substances was done by intravaginal instillation of 10 µL of each substance on day 1, 2 and 3, then every two days up to day 15. The evolution of the infection was monitored by making vaginal cultures from each animal on SDA and the number of yeast colonies counted.

### 3.3. Statistical analysis

The one way analysis of variance (One Way ANOVA) was used to analyze the number of colonies and results were expressed as mean ± standard deviation. The results were compared using the Waller-Duncan test at a 5% probability level.

## 4. Conclusions

Based on these results, one of the possible modes of action of this oil is to effect lipid biosynthesis and particularly that of fatty acids. Fatty acids are among the major building blocks of living cells, making lipid biosynthesis a potent target for antifungal substances. The essential oil of *C. ambrosioides* may be used in the treatment of vaginal candidiasis in combination with other natural antifungal substances. But further studies in the evaluation of its toxicity as well as pharmacokinetic-pharmacodynamic parameters should be envisaged.
